# Validity of two weight prediction models for community-living patients participating in a weight loss program

**DOI:** 10.1038/s41598-023-38683-9

**Published:** 2023-07-19

**Authors:** Robert Dent, Neil Harris, Carl van Walraven

**Affiliations:** 1 Department of Medicine, The Ottawa Hospital, Ottawa, Canada; 2 Weight Management Clinic, The Ottawa Hospital, Ottawa, Canada; 3 Ottawa Hospital Research Institute, Institute for Clinical Evaluative Sciences, University of Ottawa, ASB1-003 1053, Carling Ave, Ottawa, ON K1Y 4E9 Canada

**Keywords:** Obesity, Epidemiology

## Abstract

Models predicting individual body weights over time clarify patient expectations in weight loss programs. The accuracy of two commonly used weight prediction models in community living people is unclear. All eligible people entering a weight management program between 1992 and 2015 were included. Patients’ diet was 1200 kcal/day for week 0 followed by 900 kcal/day for weeks 1–7 and were excluded from the analysis if they were nonadherent. We generated expected weights using the National Institutes of Health Body Weight Planner (NIH-BWP) and the Pennington Biomedical Research Center Weight Loss Predictor (PBRC-WLP). 3703 adherent people were included (mean age 46 years, 72.6% women, mean [SD] weight 262.3 pounds [54.2], mean [SD] BMI 42.4 [7.6]). Mean (SD) relative body weight differences (100*[observed−expected]/expected) for NIH-BWP and PBRC-WLP models was − 1.5% (3.8) and − 2.9% (3.2), respectively. At week 7, mean squared error with NIH-BWP (98.8, 83%CI 89.7–108.8) was significantly lower than that with PBRC-WLP (117.7, 83%CI 112.4–123.4). Notable variation in relative weight difference were seen (for NIH-BWP, 5th–95th percentile was − 6.2%, + 3.7%; Δ 9.9%). During the first 7 weeks of a weight loss program, both weight prediction models returned expected weights that were very close to observed values with the NIH-BWP being more accurate. However, notable variability between expected and observed weights in individual patients were seen. Clinicians can monitor patients in weight loss programs by comparing their progress with these data.

## Introduction

When starting a weight loss program, realistic expectations regarding weights over time are essential to optimize compliance and identify optimal weight management strategies. Two freely available models exist that return individualized expected weights over time. The National Institutes of Health Body Weight Planner (NIH-BWP) by Hall et al.^1^ is a non-linear model with four state variables defined by glycogen stores, extracellular water, fat mass, and lean mass that returns individualized weights over time using—in its simplest form—the person’s age and sex, baseline weight and height, and daily caloric intake. The Pennington Biomedical Research Center-Weight Loss Predictor (PBRC-WLP) by Thomas et al.^2,3^ is a one-dimensional differential equation model of weight change over time using the same covariates. Both models are operationalized for individual patients through websites^4,5^.

External validations of these models, in which patient-specific differences between observed and expected weights are made, are notable for several points (Table 1). First, these weight prediction models have been tested in only a limited number of subjects (a total of less than 225 people). Second, several of these validations were conducted by the original investigators in the same publication of the original model. Third, patient cohorts in the validations had a low prevalence of higher obesity classes with mean baseline BMIs being lower than 30 in the majority of validations. Fourth, there has been only one published direct comparison of these two models including 113 people that determined daily caloric intake using the intake-balance method^6^. However, the intake-balance method can significantly underestimate daily caloric intake by a mean of 3.7% with 95% confidence intervals for patient-specific estimate difference ranging from + 368 to − 688 kcal/day (n = 35)^7^. This comparison concluded that the NIH-BWP more accurately predicted weight at 2-years, with it underestimating weights by a mean of 8.4 lbs (3.8 kg) with notable variation of errors in individualized weight predictions. Fifth, one of the validation studies^15^ was “in residence” (i.e. with most caloric intake conducted under study observation). Results from such data might differ from more common “real-world” weight loss programs in which caloric intake is not directly observed. Finally, important deviation between observed and model expected weights are seen, with short-term mean absolute errors ranging from 3.7 to 5.5 lbs (1.7–2.5 kg).

**Table 1:** Summary of studies measuring patient-specific accuracy of National Institutes of Health-Body Weight Planner (NIH-BWP) or Pennington Biomedical Research Center Weight Loss Predictor (PBRC-WLP).

Validation cohort	Valid-ation N	In resi-dence?	Mean baseline BMI	Projection length (weeks)	Model	Mean weight difference, Pounds (Mean Wt_(O-E)_)
CALERIE (Phase I)^15^	24 (3)	Y^†^	27.7	24	PBRC-WLP	+ 4.0 (SD 2.9)
Racette Study^16^	13 (3)	N	31.2	12	PBRC-WLP	− 3.7 (SD 2.6)
Overfeeding study^17^	22 (3)	N	28.5	8	PBRC-WLP	+ 5.5 (SD 4.6)
CALERIE (2-Year)^18^	113 (6)	N	25.1*	104	PBRC-WLP	− 8.4 (95%CI 7.7–9.2)
					NIH-BWP	+ 1.0 (95%CI 0.03–2.0)
Not Specified	49 (10)	N	37.4	13	NIH-BWP	NR

Data for longest follow-up time in each study is presented. ^†^All but 9 weeks were in residence. *From entire CALERIE cohort (n = 218); mean baseline BMI not reported for 113 included in this sub-analysis. NR = Not reported. This study presented means of the observed weight change (29.0 lbs, SD 19.6) and the expected weight change (30.8 lbs, SD 20.0) over time for the cohort but not mean individual-patient absolute weight differences.

External validation of any model is essential for its assessment^8^. Knowing the expected variability between observed and expected weights from weight prediction models would help clinicians monitoring patients participating in weight loss programs. The objective of this study was to directly compare observed to expected weights from these two models in a large population of people participating in a weight loss program.

## Methods

### Study setting and program

This was a cohort study approved by the Ottawa Health Science Network Research Ethics Board (OHSN-REB). All data collection and analyses were performed in accordance with OHSN-REB regulations. All patients provided written informed consent to permit the use of their data. The study used prospectively collected data that were recorded in a database^9^ for the Ottawa Hospital Bariatric Centre. This program is a 26-week intensive skill-building program for weight management consisting of weekly 3-h group sessions facilitated by registered dietitians, social workers, behaviourists, and exercise specialists. Patients paid for the program up to 2010, after which it was funded by the Ontario Ministry of Health.

The program’s first intervention (week 0) was a 1200 kcal/day diet for which participants were given detailed instructions. From weeks 1 to 7, participants consumed exclusively a program-provided meal replacement program (Optifast® 900 from Nestle Health Science, Appendix A), paid for by the participants, which provided 900 kcal/day. This study’s analysis ended after week 7 because dietary prescription, and known caloric intake, changed for some of the participants after this point.

### Study cohort and data collection

**Figure 1:**
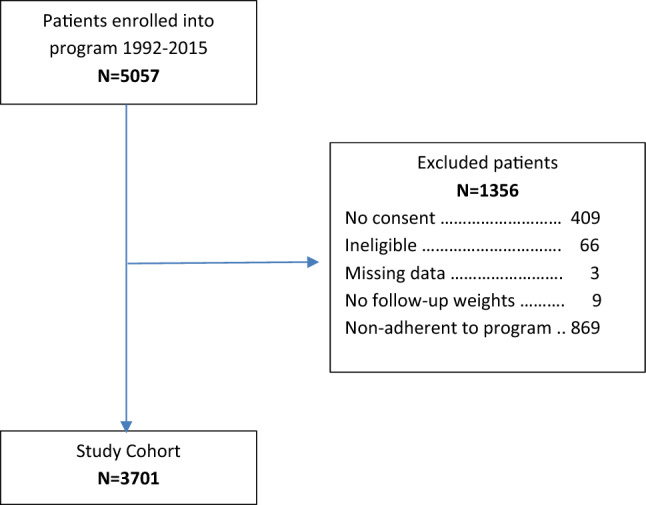
Study cohort creation.

All people who enrolled in the program between 1992 (the year that the program started) and 2015 (final year of data collection for the current wave of studies) were eligible for study inclusion (n = 5057, Fig. 1). A total of 1356 people were excluded from the current study (26.8%) because they: did not consent to their data being used for research (n = 409, 30.2%); were ineligible for weight projections with the models (because of age less than 18, weight exceeding 450 pounds [204.5 kg], or height less than 55 inches [1.4 m]) (n = 66, 4.8%); were missing variables required for the models (n = 3, 0.2%); had no follow-up weights measured at the clinic (n = 9, 0.7%), or were non-adherent to the program diet at any time during follow-up (n = 869, 64.2%).

Patient-specific baseline weight was calculated as the mean of weights taken at program intake (week − 1) and program initiation prior to any dietary intervention (week 0). All weights, including baseline and those done after each program week, were measured in the clinic between 17:00 and 20:00 with people shoeless and wearing only light clothing. Participants were deemed non-adherent at any time during the program if meal replacement usage in weeks 1–7 was < 80% or > 100% of that which had been prescribed, as documented in the weekly physician notes, or if people attended less than two thirds of the weekly meetings.

Expected weights were generated using the NIH-BWP using the online webpage calculator accessed in March 2021^4^ and PBRC-WLP using a spreadsheet program downloaded in October 2020^5^. Both models used patient age, sex, height, baseline weight, and daily caloric intake to return expected weights over time. We assumed that daily caloric intake was exclusively that from the recommended (week 0) or provided diet (weeks 1–7). NIH-BWP also requires an estimated activity level; based on clinical experience of patients in the program, and similar to a previous validation study^10^, this was defaulted to the lowest activity level for all people (i.e. a physical activity level of 1.4, described as ‘sedentary’).

### Analysis

Differences between observed and expected weights were summarized using percent relative weight differences, calculated as:

$$\frac{{100*\left( {O - \hat{E}} \right)}}{{\hat{E}}}$$

where *O* is the observed weight and $\hat{E}$ is the expected weight from the model. We quantified model error using mean squared error (MSE): ${{\sum \left( {O - \hat{E} } \right)^{2} } / n}$ where *n* is the total number of people in the analysis. To compare the accuracy of the weight prediction models, we used bootstrap methods (1000 bootstrap samples with replacement) and the percentile method^11^ to create 83% confidence intervals around the MSE from each model; this is possible because point estimates whose 83% confidence intervals do not overlap differ significantly from each other with a p-value of < 0.05^12^.

## Results

The study included 3701 people who were middle-aged (mean age 46 [SD 11.4]) and predominantly (73.5%) female (Table 2). People were heavy with a mean (SD) baseline weight and body mass index of 261.1 (53.5) pounds (118.7 [24.3] kg) and 42.3 (7.6) kg/m^2^, respectively. 3607 people (97.5%) had 5 or more follow-up body weights measured. Men were heavier and taller than the women, with a greater BMI.

**Table 2:** Description of study cohort.

Variable	Value	Females	Males	Total
		N = 2722 (73.5%)	N = 979 (26.5%)	N = 3701
Mean age (SD)		46.1 ± 11.1	48.9 ± 11.1	46.8 ± 11.2
Baseline morphological measures				
Mean weight (SD), pounds		247.2 ± 47.0	299.5 ± 47.0	261.1 ± 53.5
Mean weight (SD), kg		123.6 ± 21.4	136.1 ± 21.4	118.7 ± 24.3
Mean height (SD), inches		64.4 ± 2.5	69.7 ± 2.7	65.8 ± 3.5
Mean height (SD), m		1.64 ± 0.06	1.77 ± 0.07	1.67 ± 0.09
Mean abdominal girth (SD), inches		45.2 ± 6.0	53.5 ± 10.4	47.4 ± 8.3
Mean abdominal girth (SD), m		1.15 ± 0.15	1.36 ± 0.26	1.20 ± 0.21
Mean BMI (SD), kg/m^2^		41.9 ± 7.7	43.3 ± 7.2	42.3 ± 7.6
Number of weights during study	< 5	65 (2.4%)	29 (3.0%)	94 (2.5%)
	5	170 (6.2%)	62 (6.3%)	232 (6.3%)
	6	782 (28.7%)	245 (25.0%)	1027 (27.7%)
	7	1705 (62.6%)	643 (65.7%)	2348 (63.4%)

**Figure 2:**
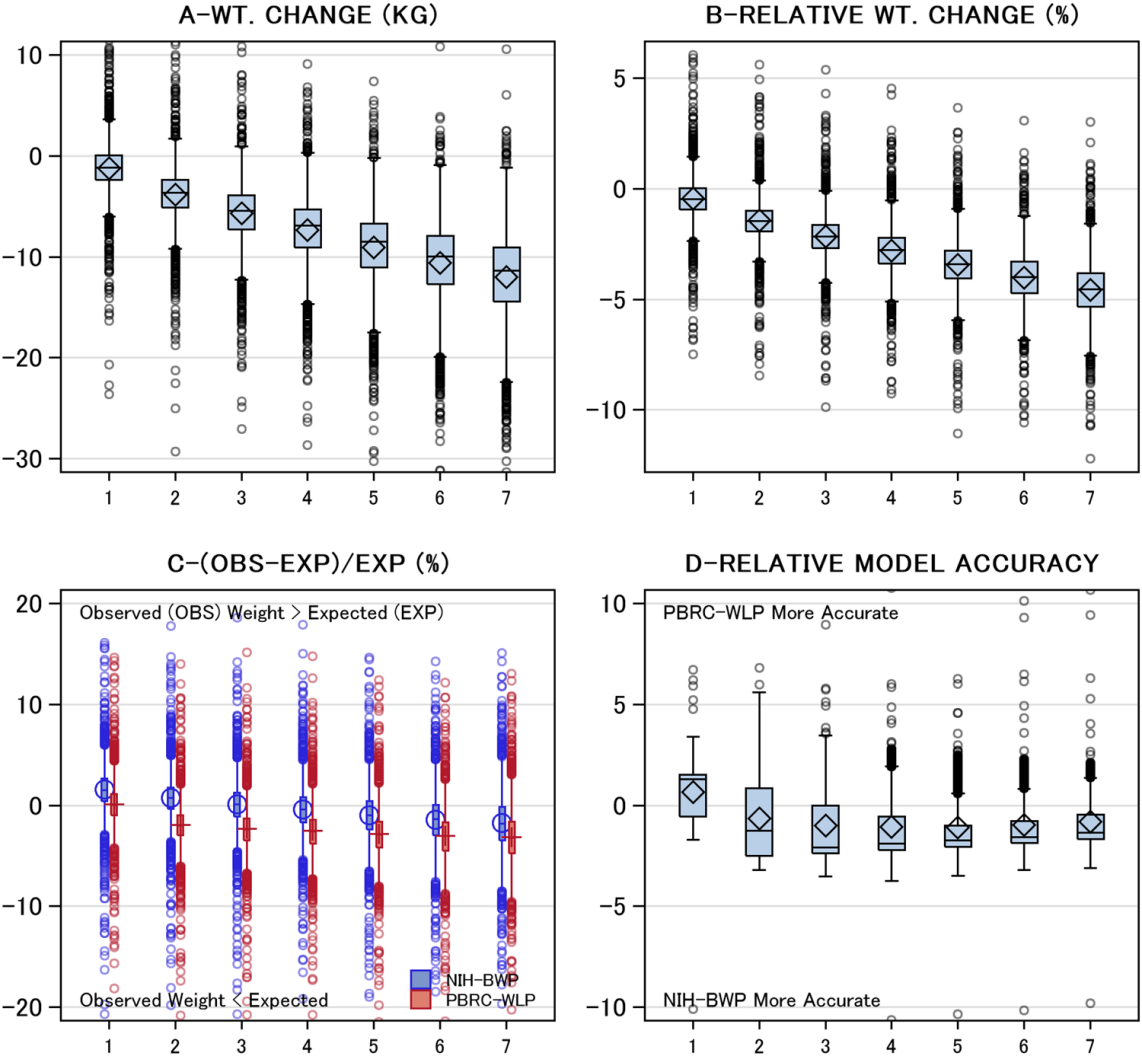
Weight changes and weight prediction model accuracy. These plots summarize changes in weight statistics (vertical axes) by program week (horizontal axis). These measures include: (**A**) Weight (WT) change in weight in pounds (LBS) from baseline; (**B**) change in weight relative to baseline (calculated as: 100*(weight−baseline)/baseline); (**C**) relative difference between observed and expected weights based on the National Institutes of Health-Body Weight Planner (blue) and the Pennington Biomedical Research Center Weight Loss Predictor (red) weight prediction models; and (**D**) relative weight prediction model accuracy, calculated as patient-specific difference in relative weight difference for the weight prediction models. All statistics are summarized using Tukey plots in which the box’s middle, lower end, and upper end indicate the median, 25th percentile, and 75th percentile, respectively, and the whiskers extend 1.5 times the interquartile range (i.e. length of box) from each end of the box. The mean and standard deviation is indicated by the diamond in each box plot.

Weight loss was extensive such that participants lost a median of 25.0 lbs (11.4 kg) with an interquartile range [IQR] of 20.0–31.8 lbs (9.1–14.4 kg) by the program’s end (Fig. 2A). Weight changes during the 7-week program was essentially linear in shape. This translated to notable relative weight changes over time with a median relative weight loss of 10.0% (IQR 8.4–11.7%) by week 7 (Fig. 2B).

The predicted body weights from each model were similar to the observed weights (Fig. 2C). Overall, both models returned expected weights that *exceeded* observed weights. Therefore, both weight prediction models tended to *under*estimate observed weight loss. This deviation between expected and observed weight increased over time. Following the first week of the program, this deviation was larger with PBRC-WLP. At the program’s end, the mean (SD) relative body weight differences for NIH-BWP and PBRC-WLP models was − 1.8% (3.5) and − 3.1% (3.1), respectively. However, differences between observed and expected weights ranged greatly for both weight prediction models with median relative differences (5th, 95th percentiles) of − 1.8% (− 6.3, 2.8) and − 3.2% (− 7.7, 1.3) for NIH-BWP and PBRC-WLP models, respectively.

Patient-specific comparison of weight prediction model accuracy confirmed that the NIH-BWP model was closer to observed weights (Fig. 2D). After week 1, body weights predicted by the NIH-BWP were closer to observed weights than weights predicted by PBRC-WLP. Mean squared errors (MSE) reflected these differences; at week 7, MSE with NIH-BWP (98.8, 83%CI 89.7–108.8) was significantly lower than that with PBRC-WLP (117.7, 83%CI 112.4–123.4).

## Discussion

To our knowledge, this is the largest external validation of the two weight prediction models most commonly used by clinicians. We found that both the National Institutes of Health Body Weight Planner (NIH-BWP) and the Pennington Biomedical Research Center Weight Loss Predictor (PBRC-WLP) returned expected short term weight changes that were very similar to observed values with the NIH-BWP being significantly more accurate. However, even after a short observation time of less than 2 months, the relative difference between observed and expected weights from the NIH-BWP ranged between − 6.3% and + 2.8% for 90% of people. Clinicians can monitor patients in weight loss programs by comparing their progress with these data.

Our analysis highlights three important points. First, these freely available models reliably predict individualized weight over time as a function of patient age, sex, height, weight, and caloric intake. Similar to a previous study^6^, we found that the NIH-BWP was significantly more accurate at predicting weight over time in patients. Although differences between these two models were small in our study, they were statistically significant and could likely increase in size over time. Therefore, these data suggest that clinicians needing a model to forecast patient weight over time should use the NIH-BWP. Second, notable variation between observed and expected body weights for individual patients existed. In addition to more accurate energy intake and expenditure measurement, we wonder if some of this variation might be resolved by considering other patient level covariates when predicting weights. It is possible that accounting for genetic, endocrinological, pharmaceutical and behavioral factors that influence weight loss might improve weight change prediction^13,14^. Finally, our data give clinicians tools to better monitor patients in weight loss programs. We prospectively collected weight data over time in a large cohort of adherent patients consuming a defined daily caloric intake. This let us describe distributions of observed weight loss relative to that predicted by weight prediction models (Fig. 2C). By comparing patient weights to these distributions, clinicians could determine how responsive they are to weight loss and whether adherence might be an issue.

Several factors should be kept in mind when reviewing our results. First, although this is a large cohort of real-world patients, it is a single-center study. It would be ideal if this analysis could be replicated using other data. Second, a primary advantage of our analysis is the precise measure of the caloric intake for adherent individuals in weeks 1–7. However, because this meal replacement program lasted a short time, our observation period is notably shorter than all other validation studies that have been done (Table 1). As a result, the differences between observed and model-expected weights seen in our analysis were small (Fig. 2A). Of course, these differences would increase if follow-up time would increase. In addition, our calculations assumed that patients consumed exclusively the recommended diets or provided supplements. Other caloric intake—or, rarely, incomplete ingestion of the meal plan supplements—would introduce error in the predicted weights. Lastly, the NIH-BWP incorporates exertion into its calculations. In our experience, most of our patients are very sedentary so we defaulted all patient “physical exertion levels” for the NIH-BWP calculation to “sedentary”. It is possible that NIH-BWP accuracy would have increased in our study more precise estimates of patient exertion over time.

In summary, clinicians and researchers can confidently use commonly available weight prediction models to forecast weights for individual patients with the NIH-BWP being more accurate. However, notable variation is seen between observed and expected body weights over time. Further research is required to determine whether notable variabilities between observed and expected weights can be explained by other covariates.

## Supplementary Information

Supplementary Information.

## Data Availability

The datasets used and/or analysed during the current study available from the corresponding author on reasonable request.
