# Supplementary material for: Validity of two weight prediction models for community-living patients participating in a weight loss program
Source: Sci Rep. 2023 Jul 19;13:11629. doi: 10.1038/s41598-023-38683-9 (PMC10356859; doi:10.1038/s41598-023-38683-9)
Supplement: Supplementary file 1 — Supplementary Information. [file 41598_2023_38683_MOESM1_ESM.docx]

**APPENDIX A:**  Composition of each Optifast 900 packet.

- Proteins: 22.5 g
- Carbohydrates 18.8 g,
- Fats 7.5 g,
- Electrolytes: sodium 450 mg, Potassium: 570 mg, Chloride 415 mg
- Vitamins and minerals: each packet contained >25% Health Canada and FDA daily requirements (except vitamin K).
- Energy: 225 kcal.

Each packet was mixed with 300 mL water. People consumed 4 packets per day to provide 900 kcal.
